# Clinical pregnancy, live birth, and twin pregnancy outcomes of nine frozen-thawed embryo transfer strategies across female age groups: a real-world cohort study

**DOI:** 10.3389/fendo.2026.1890802

**Published:** 2026-09-01

**Authors:** Lei Sun, Jingjing Huang, Liping Huang, Tian Hu, Weiwei Deng, Hongbo Wu

**Affiliations:** Center for Reproductive Medicine, Qinzhou Maternal and Child Health Hospital, Qinzhou, China

**Keywords:** clinical pregnancy, embryo transfer strategy, female age, frozen-thawed embryo transfer, live birth, twin pregnancy

## Abstract

**Background:**

The selection of a frozen-thawed embryo transfer (FET) strategy requires balancing the potential for improved clinical pregnancy and live birth outcomes against the risk of multiple pregnancy. However, comprehensive evaluations of transfer strategies defined by embryo developmental stage (day 3 [D3] cleavage-stage embryos, day 5 [D5] blastocysts, or day 6 [D6] blastocysts), embryo quality (high-quality or non-high-quality usable embryos), and the number of embryos transferred (single or double), particularly in relation to the potential modifying effect of female age, remain limited. In addition, real-world evidence is insufficient to guide clinical decision-making in common scenarios involving patients who have both cleavage-stage embryos and blastocysts available or those who have only D3 cleavage-stage embryos without blastocyst culture.

**Methods:**

We retrospectively analyzed eligible FET cycles performed at our center between January 2024 and December 2025. Patients were stratified into four age groups: <30, 30–34, 35–39, and ≥40 years. Clinical pregnancy, live birth, and twin pregnancy outcomes were compared across nine embryo transfer strategies. The potential modifying effect of female age on the associations between transfer strategy and clinical pregnancy or live birth was further evaluated.

**Results:**

Using D5 single high-quality blastocyst transfer as the reference, D5 double high-quality blastocyst transfer was associated with higher odds of clinical pregnancy and live birth, but also with a markedly increased per-transfer-cycle risk of twin pregnancy. D5 double blastocyst transfer comprising one high-quality and one usable blastocyst showed no clear advantage in clinical pregnancy or live birth but was associated with an increased per-transfer-cycle risk of twin pregnancy. D5 single usable blastocyst transfer, D3 single high-quality cleavage-stage embryo transfer, and sequential transfer of two usable embryos were associated with lower odds of clinical pregnancy and/or live birth. D3 double high-quality cleavage-stage embryo transfer was associated with lower odds of clinical pregnancy only, whereas D6 single high-quality blastocyst transfer did not differ significantly from the reference strategy. The adjusted probabilities of clinical pregnancy and live birth generally declined with increasing female age across all transfer strategies. However, no statistically significant age-by-strategy interaction was observed, providing insufficient evidence to support fixed transfer strategies based solely on female age.

**Conclusions:**

Clinical pregnancy, live birth, and per-transfer-cycle twin pregnancy risk differed across FET strategies. D5 single high-quality blastocyst transfer generally showed favorable reproductive outcomes and a relatively low risk of twin pregnancy and may therefore serve as a clinically relevant reference strategy. D5 double high-quality blastocyst transfer may increase the probability of clinical pregnancy and live birth within a single transfer cycle but is accompanied by a substantially higher risk of twin pregnancy. The current findings do not support selecting a fixed transfer strategy based solely on female age. Clinical decision-making should integrate embryo developmental stage, embryo quality, the number of embryos transferred, baseline patient prognosis, and the risk of multiple pregnancy.

## 1 Introduction

Frozen-thawed embryo transfer (FET) has become one of the most widely used treatment approaches in assisted reproductive technology (ART) (1, 2). A key consideration in selecting an FET strategy is how to improve the probability of pregnancy within a single transfer cycle while minimizing the risk of multiple pregnancy, particularly twin pregnancy, and its associated obstetric complications and adverse perinatal outcomes (3, 4). Double-embryo transfer may increase the pregnancy rate per transfer cycle but also substantially increases the risk of twin pregnancy, which is closely associated with preterm birth, low birth weight, and maternal complications. By contrast, single-embryo transfer can reduce the risk of twin pregnancy, although its reproductive outcomes may be influenced by factors such as female age and embryo quality (1, 3, 4).

Embryo-related factors affecting FET outcomes can be broadly categorized into three dimensions (1): embryo developmental stage, namely the choice between day-3 (D3) cleavage-stage embryos and day-5 (D5) or day-6 (D6) blastocysts (2); embryo quality, including the distinction between high-quality embryos and non-high-quality usable embryos; and (3) the number of embryos transferred, involving the balance between single- and double-embryo transfer (4–7). Although these individual factors have been widely investigated, large-scale real-world studies systematically comparing the combined transfer strategies derived from these three dimensions across different female age groups remain limited (8).

In clinical practice, two common decision-making scenarios remain insufficiently supported by evidence. First, when both cleavage-stage embryos and blastocysts are available, the optimal transfer strategy remains uncertain. Previous studies have suggested that blastocyst transfer may reduce the risk of ectopic pregnancy (0.8% vs 1.8%) and that single-blastocyst transfer may maintain favorable implantation potential while reducing the risk of multiple pregnancy (9, 10). Second, for women of advanced reproductive age with diminished ovarian reserve, a limited number of embryos, and only cryopreserved D3 cleavage-stage embryos without blastocyst culture, the appropriate number of embryos to transfer remains unclear. Previous evidence suggests that double cleavage-stage embryo transfer in women aged ≥38 years may improve the probability of pregnancy to some extent, although the risk of multiple pregnancy must also be considered (3). Another study indicated that thawing cryopreserved D3 embryos and continuing culture to the blastocyst stage before transfer may result in better pregnancy outcomes than direct D3 embryo transfer (11).

Using real-world FET data from our center, this study compared transfer strategies defined by embryo developmental stage, morphological quality, and number of embryos transferred across different female age groups. We specifically evaluated the associations of these strategies with clinical pregnancy, live birth, and twin pregnancy outcomes in the above-mentioned clinical scenarios, with the aim of providing evidence to support individualized selection of FET strategies.

## 2 Materials and methods

### 2.1 Study design and population

We retrospectively screened frozen-thawed embryo transfer (FET) cycles performed at the Center for Reproductive Medicine, Qinzhou Maternal and Child Health Hospital, between January 2024 and December 2025. For patients with more than one eligible FET cycle during the study period, only the first eligible cycle was included.

**Figure 1:**
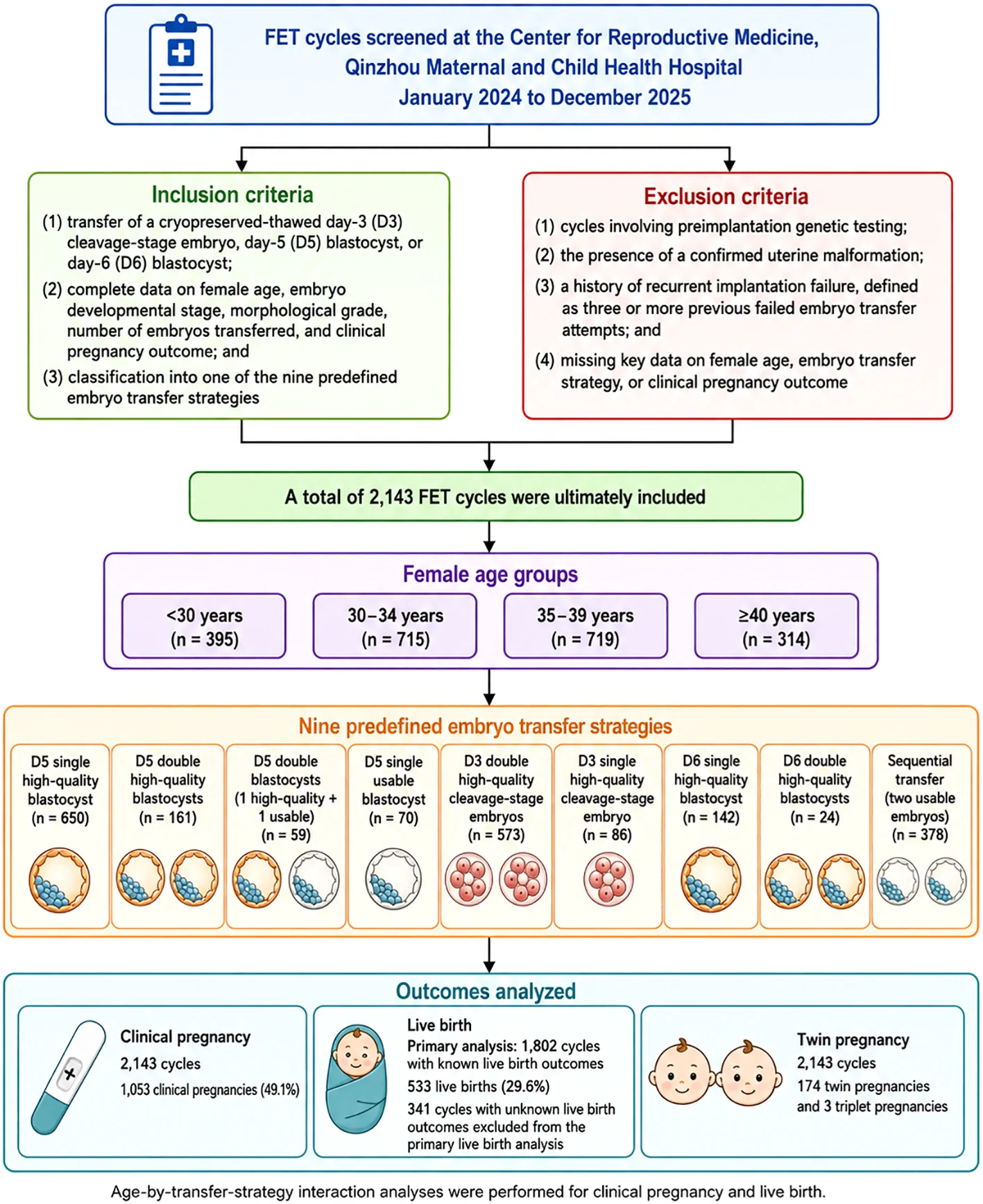
Study flowchart.

The inclusion criteria were as follows (1): transfer of a cryopreserved-thawed day-3 (D3) cleavage-stage embryo, day-5 (D5) blastocyst, or day-6 (D6) blastocyst (2); complete data on female age, embryo developmental stage, morphological grade, number of embryos transferred, and clinical pregnancy outcome; and (3) classification into one of the nine predefined embryo transfer strategies. The exclusion criteria were as follows (1): cycles involving preimplantation genetic testing (2); the presence of a confirmed uterine malformation (3); a history of recurrent implantation failure, defined as three or more previous failed embryo transfer attempts; and (4) missing key data on female age, embryo transfer strategy, or clinical pregnancy outcome. A total of 2,143 FET cycles were ultimately included (**Figure 1**).

This retrospective cohort study was approved by the Ethics Committee of Qinzhou Maternal and Child Health Hospital (Ethics No. QSFYL〔2026〕05002). The requirement for written informed consent was waived because only de-identified data obtained during routine clinical care were retrospectively analyzed.

### 2.2 Embryo culture, grading, and outcome definitions

Day-3 cleavage-stage embryos were evaluated according to the consensus criteria for morphological assessment of human embryos (5). High-quality D3 embryos were defined as embryos with ≥7 blastomeres, <20% fragmentation, and symmetrical blastomeres. Usable D3 embryos were defined as embryos with ≥4 blastomeres and <50% fragmentation. Blastocysts were evaluated using the Gardner grading system (6, 7). High-quality blastocysts were defined as those with an expansion grade of ≥3 and with both the inner cell mass (ICM) and trophectoderm (TE) graded A or B. Usable blastocysts were defined as those with an expansion grade of ≥2 and at least one C grade for the ICM or TE.

Clinical pregnancy was defined as the presence of an intrauterine gestational sac with detectable fetal cardiac activity on transvaginal ultrasonography 6–7 weeks after embryo transfer. Live birth was defined as the delivery of at least one live-born infant resulting from the index pregnancy. Cycles without pregnancy, with biochemical pregnancy, with a documented spontaneous miscarriage, or with stillbirth only were coded as no live birth. Cycles in which a clinical pregnancy was achieved but no live birth, miscarriage, stillbirth, or delivery outcome was documented were classified as having an unknown live birth outcome. A total of 341 such cycles were excluded from the primary live birth analysis. Twin pregnancy was defined as the ultrasonographic detection of two gestational sacs, both with fetal cardiac activity, during a clinical pregnancy cycle. Triplet pregnancy was defined as the ultrasonographic detection of three gestational sacs, all with fetal cardiac activity. The twin pregnancy rate among clinical pregnancies was calculated using clinical pregnancy cycles as the denominator, whereas the per-transfer-cycle risk of twin pregnancy was calculated using all transfer cycles as the denominator.

### 2.3 Embryo transfer strategies and female age groups

Nine embryo transfer strategies were defined according to embryo developmental stage or transfer type—D3 cleavage-stage embryo, D5 blastocyst, D6 blastocyst, or sequential transfer—embryo quality—high-quality or non-high-quality usable embryo—and the number of embryos transferred—single or double transfer—as detailed in **Table 1**. Female age was categorized into four groups: <30, 30–34, 35–39, and ≥40 years.

**Table 1:** Definitions and composition of the nine frozen-thawed embryo transfer strategies.

Embryo transfer strategy	Embryo composition	Operational definition and representative morphologic examples from the database	Sample size, n
D5 single high-quality blastocyst	Transfer of one D5 blastocyst	The blastocyst met the high-quality criteria: expansion grade ≥3, with both the inner cell mass (ICM) and trophectoderm (TE) graded A or B. Morphologies observed in the database included 3AB, 3BB, 4AA, 4AB, 4BA, 4BB, 5BA, and 5BB.	650
D5 double high-quality blastocysts	Transfer of two D5 blastocysts	Both blastocysts met the high-quality criteria described above. Common combinations in the database included 4BB + 4BB, 4BB + 3BB, and 3BB + 3BB.	161
D5 double blastocysts (1 high-quality + 1 usable)	Transfer of two D5 blastocysts	The transfer comprised one high-quality blastocyst and one non-high-quality usable blastocyst. The latter had an expansion grade ≥2 and at least one C grade for the ICM or TE. Observed combinations included 4BB + 4CB, 4BB + 4BC, and 3BB + 3BC.	59
D5 single usable blastocyst	Transfer of one D5 blastocyst	The blastocyst was a non-high-quality usable blastocyst with an expansion grade ≥2 and at least one C grade for the ICM or TE. Morphologies observed in the database included 3BC, 3CB, 4AC, 4BC, 4CA, and 4CB.	70
D3 double high-quality cleavage-stage embryos	Transfer of two D3 cleavage-stage embryos	Both embryos met the criteria for high-quality D3 embryos: ≥7 blastomeres, <20% fragmentation, and symmetrical blastomeres. Common combinations in the database included grade II 8-cell + grade II 8-cell embryos and grade I 8-cell + grade II 8-cell embryos.	573
D3 single high-quality cleavage-stage embryo	Transfer of one D3 cleavage-stage embryo	The embryo met the criteria for a high-quality D3 embryo: ≥7 blastomeres, <20% fragmentation, and symmetrical blastomeres. Common morphologies in the database included grade II 8-cell, grade II 7-cell, and grade I 8-cell embryos.	86
D6 single high-quality blastocyst	Transfer of one D6 blastocyst	The blastocyst met the high-quality criteria: expansion grade ≥3, with both the ICM and TE graded A or B. Morphologies observed in the database included 3AB, 3BB, 4AB, 4BA, 4BB, 5BA, 5BB, and 6BB.	142
D6 double high-quality blastocysts	Transfer of two D6 blastocysts	Both blastocysts met the high-quality criteria described above. Observed combinations included 4BB + 4BB, 4BB + 3BB, and 4BA + 4BB.	24
Sequential transfer (two usable embryos)	Sequential transfer of two embryos within the same frozen-thawed embryo transfer cycle	One D3 cleavage-stage embryo was transferred first, followed by one D5 or D6 blastocyst (D3/D5 or D3/D6 sequential transfer). Both embryos met the applicable transferability criteria: the D3 embryo had ≥4 blastomeres and <50% fragmentation; the blastocyst had an expansion grade ≥2 with at least one C grade for the ICM or TE, or met the high-quality blastocyst criteria.	378

D3, D5, and D6 denote days 3, 5, and 6 of embryo development, respectively. Blastocysts were graded using the Gardner scoring system; the numeral indicates the degree of expansion, and the first and second letters indicate the quality of the inner cell mass (ICM) and trophectoderm (TE), respectively. In the “1 high-quality + 1 usable” group, “usable” specifically refers to a non-high-quality usable blastocyst. In the sequential-transfer group, “two usable embryos” refers broadly to two embryos meeting the relevant transferability criteria. The total sample comprised 2,143 transfer cycles, and all morphologic examples were derived from the study database.

### 2.4 Statistical analysis

All continuous variables were non-normally distributed and are presented as medians with first and third quartiles, median (Q1, Q3). Comparisons among groups were performed using the Kruskal–Wallis H test. Categorical variables are presented as numbers and percentages, n (%). The Pearson chi-square test was used when the sample size and expected cell counts were adequate; otherwise, Fisher’s exact test was applied. Logistic regression models were used to evaluate the associations of embryo transfer strategy with clinical pregnancy and live birth, with results reported as odds ratios (ORs), adjusted odds ratios (aORs), and 95% confidence intervals (CIs). Because twin pregnancy events were relatively uncommon and no events occurred in some transfer-strategy groups, Firth penalized logistic regression was used to analyze the risk of twin pregnancy. Potential effect modification by female age was evaluated by including an age-by-transfer-strategy interaction term in the regression models. Sensitivity analyses were performed to assess the robustness of the findings. All tests were two-sided, and a P value <0.05 was considered statistically significant. Statistical analyses were conducted using SPSS version 26.0 and R version 4.4.2.

## 3 Results

### 3.1 Study population and baseline characteristics

Female age, male age, body mass index (BMI), duration of infertility, anti-Müllerian hormone (AMH) level, antral follicle count (AFC), number of oocytes retrieved, number of usable embryos, and endometrial thickness on the day of transfer differed significantly across the four female age groups (all *P*<0.05). With increasing age, AMH, AFC, the number of oocytes retrieved, and the number of usable embryos generally decreased, whereas BMI showed an overall increasing trend. The distributions of infertility factor category, polycystic ovary syndrome (PCOS), tubal/pelvic factors, ovulatory dysfunction, diminished ovarian reserve, fertilization method, and endometrial preparation regimen also differed significantly across age groups (all *P*<0.05). No statistically significant between-group difference was observed for endometriosis/uterine factors (*P*=0.075) (**Table 2**).

**Table 2:** Baseline and cycle characteristics of patients across female age groups.

Variable	Category	Overall (n = 2,143)	<30 years (n = 395)	30–34 years (n = 715)	35–39 years (n = 719)	≥40 years (n = 314)	Test statistic	P value
Female age, years		34.00 (31.00, 38.00)	28.00 (26.00, 29.00)	32.00 (31.00, 33.00)	37.00 (36.00, 38.00)	41.00 (40.00, 43.00)	H=1968.47	<0.001
Male age, years		36.00 (32.00, 39.00)	30.00 (28.00, 33.00)	34.00 (32.00, 36.00)	38.00 (36.00, 40.00)	42.00 (39.00, 45.00)	H=1134.00	<0.001
Body mass index		22.27 (20.40, 24.78)	21.50 (19.55, 23.78)	22.03 (20.03, 24.65)	22.49 (20.70, 24.77)	23.63 (21.89, 26.04)	H=79.21	<0.001
Duration of infertility, years		3.00 (1.00, 5.00)	3.00 (1.00, 4.00)	3.00 (1.00, 5.00)	3.00 (1.00, 6.00)	3.00 (1.00, 6.00)	H=15.79	0.001
AMH		3.59 (2.16, 6.11)	4.95 (3.30, 7.41)	4.29 (2.79, 7.35)	3.13 (1.93, 5.37)	1.81 (1.02, 3.16)	H=331.57	<0.001
AFC		16.00 (11.00, 24.00)	21.00 (15.00, 27.00)	19.00 (13.00, 26.00)	14.00 (9.00, 20.00)	10.00 (6.00, 14.00)	H=355.98	<0.001
Number of oocytes retrieved		19.00 (12.50, 26.00)	23.00 (18.00, 29.00)	21.00 (15.00, 28.00)	16.00 (11.00, 23.00)	12.00 (8.00, 19.00)	H=284.58	<0.001
Number of usable embryos		11.00 (7.00, 15.00)	13.00 (10.00, 17.50)	12.00 (9.00, 16.00)	10.00 (7.00, 14.00)	7.00 (5.00, 11.00)	H=211.84	<0.001
Endometrial thickness on transfer day		8.90 (8.00, 10.00)	9.00 (8.00, 9.95)	9.00 (8.00, 10.00)	8.80 (8.00, 10.00)	8.50 (7.93, 10.00)	H=12.63	0.006
Infertility factor category	Male factor	144 (6.7)	37 (9.4)	53 (7.4)	51 (7.1)	3 (1.0)	χ²=22.53	<0.001
	Female factor	590 (27.5)	102 (25.8)	202 (28.3)	192 (26.7)	94 (29.9)		
	Combined factors	1409 (65.7)	256 (64.8)	460 (64.3)	476 (66.2)	217 (69.1)		
PCOS	No	1762 (82.2)	274 (69.4)	563 (78.7)	619 (86.1)	306 (97.5)	χ²=107.77	<0.001
	Yes	381 (17.8)	121 (30.6)	152 (21.3)	100 (13.9)	8 (2.5)		
Tubal/pelvic factor	No	498 (23.2)	73 (18.5)	139 (19.4)	141 (19.6)	145 (46.2)	χ²=108.73	<0.001
	Yes	1645 (76.8)	322 (81.5)	576 (80.6)	578 (80.4)	169 (53.8)		
Ovulatory dysfunction	No	2062 (96.2)	379 (95.9)	676 (94.5)	695 (96.7)	312 (99.4)	χ²=14.51	0.002
	Yes	81 (3.8)	16 (4.1)	39 (5.5)	24 (3.3)	2 (0.6)		
Diminished ovarian reserve	No	1946 (90.8)	394 (99.7)	686 (95.9)	642 (89.3)	224 (71.3)	χ²=204.99	<0.001
	Yes	197 (9.2)	1 (0.3)	29 (4.1)	77 (10.7)	90 (28.7)		
Endometriosis/uterine factor	No	2032 (94.8)	377 (95.4)	687 (96.1)	678 (94.3)	290 (92.4)	χ²=6.92	0.075
	Yes	111 (5.2)	18 (4.6)	28 (3.9)	41 (5.7)	24 (7.6)		
Fertilization method	Other	183 (8.5)	45 (11.4)	58 (8.1)	49 (6.8)	31 (9.9)	χ²=55.17	<0.001
	IVF	1197 (55.9)	197 (49.9)	370 (51.7)	440 (61.2)	190 (60.5)		
	ICSI	451 (21.0)	77 (19.5)	153 (21.4)	145 (20.2)	76 (24.2)		
	IVF+ICSI	312 (14.6)	76 (19.2)	134 (18.7)	85 (11.8)	17 (5.4)		
Endometrial preparation regimen	Ovulation induction cycle	338 (15.8)	58 (14.7)	119 (16.6)	121 (16.8)	40 (12.7)	χ²=65.40	<0.001
	Hormone replacement therapy cycle	1224 (57.1)	265 (67.1)	440 (61.5)	371 (51.6)	148 (47.1)		
	Natural cycle	581 (27.1)	72 (18.2)	156 (21.8)	227 (31.6)	126 (40.1)		

This table includes 2,143 cycles assigned to the nine prespecified embryo transfer strategies. Continuous variables were non-normally distributed and are presented as median (Q1, Q3); comparisons were performed using the Kruskal-Wallis H test. Categorical variables are presented as n (%) and were compared using the Pearson chi-square test. A two-sided P < 0.05 was considered statistically significant. Q1, first quartile; Q3, third quartile; AMH, anti-Müllerian hormone; AFC, antral follicle count; IVF, in vitro fertilization; ICSI, intracytoplasmic sperm injection; PCOS, polycystic ovary syndrome.

### 3.2 Age-stratified clinical pregnancy and live birth outcomes

Among the 2,143 transfer cycles, 1,053 resulted in clinical pregnancy, corresponding to an overall clinical pregnancy rate of 49.1%. Among the 1,802 cycles with known live birth outcomes, 533 resulted in live birth, yielding a live birth rate of 29.6%. Within each of the four age groups (<30, 30–34, 35–39, and ≥40 years), the overall clinical pregnancy rates differed significantly across the nine transfer strategies. Significant between-strategy differences in live birth rates were observed in the 35–39-year and ≥40-year groups (*P*=0.002 and *P*=0.043, respectively), whereas no significant differences were observed in the younger age groups (both *P*>0.05) (**Table 3**).

**Table 3:** Clinical pregnancy and live birth outcomes by embryo transfer strategy and female age group.

Age group	Embryo transfer strategy	Total cycles, n	Clinical pregnancy, n/N (%)	95% CI(%)	P value	Known live birth outcomes, n	Live birth, n/N (%)	95% CI(%)	Miscarriage	Stillbirth	Unknown live birth outcome, n	P value
<30 years	D5 single high-quality blastocyst	173	111/173 (64.2)	56.8–70.9	0.013	126	56/126 (44.4)	36.1–53.2	8	0	47	0.259
	D5 double high-quality blastocysts	25	17/25 (68.0)	48.4–82.8		17	8/17 (47.1)	26.2–69.0	1	0	8	
	D5 double blastocysts (1 high-quality + 1 usable)	8	6/8 (75.0)	40.9–92.9		6	2/6 (33.3)	9.7–70.0	1	1	2	
	D5 single usable blastocyst	17	6/17 (35.3)	17.3–58.7		15	3/15 (20.0)	7.0–45.2	1	0	2	
	D3 double high-quality cleavage-stage embryos	78	41/78 (52.6)	41.6–63.3		69	26/69 (37.7)	27.2–49.5	6	0	9	
	D3 single high-quality cleavage-stage embryo	6	1/6 (16.7)	3.0–56.4		6	1/6 (16.7)	3.0–56.4	0	0	0	
	D6 single high-quality blastocyst	31	16/31 (51.6)	34.8–68.0		24	7/24 (29.2)	14.9–49.2	2	0	7	
	D6 double high-quality blastocysts	8	7/8 (87.5)	52.9–97.8		6	3/6 (50.0)	18.8–81.2	2	0	2	
	Sequential transfer (two usable embryos)	49	23/49 (46.9)	33.7–60.6		40	10/40 (25.0)	14.2–40.2	4	0	9	
30–34 years	D5 single high-quality blastocyst	240	145/240 (60.4)	54.1–66.4	0.005	189	75/189 (39.7)	33.0–46.8	19	0	51	0.138
	D5 double high-quality blastocysts	52	34/52 (65.4)	51.8–76.8		45	18/45 (40.0)	27.0–54.5	9	0	7	
	D5 double blastocysts (1 high-quality + 1 usable)	23	15/23 (65.2)	44.9–81.2		21	10/21 (47.6)	28.3–67.6	3	0	2	
	D5 single usable blastocyst	22	8/22 (36.4)	19.7–57.0		20	5/20 (25.0)	11.2–46.9	1	0	2	
	D3 double high-quality cleavage-stage embryos	170	73/170 (42.9)	35.7–50.5		156	45/156 (28.8)	22.3–36.4	14	0	14	
	D3 single high-quality cleavage-stage embryo	18	8/18 (44.4)	24.6–66.3		16	5/16 (31.2)	14.2–55.6	1	0	2	
	D6 single high-quality blastocyst	60	31/60 (51.7)	39.3–63.8		44	12/44 (27.3)	16.3–41.8	3	0	16	
	D6 double high-quality blastocysts	8	6/8 (75.0)	40.9–92.9		7	4/7 (57.1)	25.0–84.2	1	0	1	
	Sequential transfer (two usable embryos)	122	60/122 (49.2)	40.5–57.9		95	26/95 (27.4)	19.4–37.1	7	0	27	
35–39 years	D5 single high-quality blastocyst	198	106/198 (53.5)	46.6–60.3	<0.001	157	43/157 (27.4)	21.0–34.8	22	0	41	0.002
	D5 double high-quality blastocysts	66	47/66 (71.2)	59.4–80.7		53	27/53 (50.9)	37.9–63.9	7	0	13	
	D5 double blastocysts (1 high-quality + 1 usable)	21	12/21 (57.1)	36.5–75.5		20	9/20 (45.0)	25.8–65.8	2	0	1	
	D5 single usable blastocyst	24	8/24 (33.3)	18.0–53.3		24	5/24 (20.8)	9.2–40.5	3	0	0	
	D3 double high-quality cleavage-stage embryos	192	88/192 (45.8)	38.9–52.9		167	51/167 (30.5)	24.1–37.9	11	1	25	
	D3 single high-quality cleavage-stage embryo	32	9/32 (28.1)	15.6–45.4		29	3/29 (10.3)	3.6–26.4	3	0	3	
	D6 single high-quality blastocyst	39	13/39 (33.3)	20.6–49.0		33	5/33 (15.2)	6.7–30.9	2	0	6	
	D6 double high-quality blastocysts	8	3/8 (37.5)	13.7–69.4		7	2/7 (28.6)	8.2–64.1	0	0	1	
	Sequential transfer (two usable embryos)	139	69/139 (49.6)	41.5–57.8		115	31/115 (27.0)	19.7–35.7	14	0	24	
≥40 years	D5 single high-quality blastocyst	39	16/39 (41.0)	27.1–56.6	0.028	34	8/34 (23.5)	12.4–40.0	3	0	5	0.043
	D5 double high-quality blastocysts	18	10/18 (55.6)	33.7–75.4		16	6/16 (37.5)	18.5–61.4	2	0	2	
	D5 double blastocysts (1 high-quality + 1 usable)	7	3/7 (42.9)	15.8–75.0		6	1/6 (16.7)	3.0–56.4	1	0	1	
	D5 single usable blastocyst	7	2/7 (28.6)	8.2–64.1		7	0/7 (0.0)	0.0–35.4	2	0	0	
	D3 double high-quality cleavage-stage embryos	133	36/133 (27.1)	20.2–35.2		126	15/126 (11.9)	7.3–18.7	14	0	7	
	D3 single high-quality cleavage-stage embryo	30	4/30 (13.3)	5.3–29.7		30	1/30 (3.3)	0.6–16.7	3	0	0	
	D6 single high-quality blastocyst	12	4/12 (33.3)	13.8–60.9		11	2/11 (18.2)	5.1–47.7	1	0	1	
	D6 double high-quality blastocysts	0	—	—		0	—	—	0	0	0	
	Sequential transfer (two usable embryos)	68	15/68 (22.1)	13.8–33.3		65	8/65 (12.3)	6.4–22.5	4	0	3	

Clinical pregnancy rates were calculated using all transfer cycles within each age group and transfer strategy as the denominator. Live birth was defined as the delivery of at least one live-born infant. Cycles without pregnancy, with biochemical pregnancy, with a documented miscarriage, or with stillbirth only were coded as no live birth. Cycles with a clinical pregnancy but without a documented live birth, miscarriage, stillbirth, or delivery outcome were classified as having an unknown live birth outcome and were excluded from the live birth denominator. The 95% CIs were calculated using the Wilson method. Overall comparisons among the nine strategies within each age group were performed using the Fisher-Freeman-Halton exact test with 200,000 Monte Carlo repetitions. A total of 2,143 cycles were included; no D6 double high-quality blastocyst transfers occurred in women aged ≥40 years.

D5 double high-quality blastocyst transfer showed relatively high clinical pregnancy and live birth rates in most age groups. In the D5 single high-quality blastocyst group, the clinical pregnancy rate decreased from 64.2% among women aged <30 years to 41.0% among those aged ≥40 years, while the corresponding live birth rate decreased from 44.4% to 23.5% (**Table 3**).

### 3.3 Age-stratified twin pregnancy outcomes

A total of 174 twin pregnancies and three triplet pregnancies were observed among all included cycles. The twin pregnancy rate among clinical pregnancies was 16.5%, whereas the per-transfer-cycle risk of twin pregnancy was 8.1%. In the <30-, 30–34-, and 35–39-year groups, both the twin pregnancy rate among clinical pregnancies and the per-transfer-cycle twin pregnancy risk differed significantly across transfer strategies (all *P*<0.001). Among women aged ≥40 years, the overall difference in the twin pregnancy rate among clinical pregnancies did not reach statistical significance (*P*=0.063), whereas the between-strategy difference in per-transfer-cycle twin pregnancy risk remained statistically significant (*P*=0.007) (**Table 4**). Across all four age groups, the per-transfer-cycle twin pregnancy risk was lower with D5 single high-quality blastocyst transfer than with D5 double high-quality blastocyst transfer. Overall, twin pregnancy rates among clinical pregnancies were higher with double-embryo transfer strategies and lower with single-embryo transfer strategies (**Table 4**).

**Table 4:** Twin pregnancy outcomes by embryo transfer strategy and female age group.

Age group	Embryo transfer strategy	Total cycles, n	Clinical pregnancies, n	Twin pregnancy rate among clinical pregnancies, n/N (%)	95% CI(%)	P value	Triplet pregnancies, n	Twin pregnancy risk per transfer cycle, n/N (%)	95% CI(%)	P value
<30 years	D5 single high-quality blastocyst	173	111	1/111 (0.9)	0.2–4.9	<0.001	0	1/173 (0.6)	0.1–3.2	<0.001
	D5 double high-quality blastocysts	25	17	8/17 (47.1)	26.2–69.0		0	8/25 (32.0)	17.2–51.6	
	D5 double blastocysts (1 high-quality + 1 usable)	8	6	1/6 (16.7)	3.0–56.4		0	1/8 (12.5)	2.2–47.1	
	D5 single usable blastocyst	17	6	0/6 (0.0)	0.0–39.0		0	0/17 (0.0)	0.0–18.4	
	D3 double high-quality cleavage-stage embryos	78	41	15/41 (36.6)	23.6–51.9		1	15/78 (19.2)	12.0–29.3	
	D3 single high-quality cleavage-stage embryo	6	1	0/1 (0.0)	0.0–79.3		0	0/6 (0.0)	0.0–39.0	
	D6 single high-quality blastocyst	31	16	2/16 (12.5)	3.5–36.0		0	2/31 (6.5)	1.8–20.7	
	D6 double high-quality blastocysts	8	7	3/7 (42.9)	15.8–75.0		0	3/8 (37.5)	13.7–69.4	
	Sequential transfer (two usable embryos)	49	23	5/23 (21.7)	9.7–41.9		1	5/49 (10.2)	4.4–21.8	
30–34 years	D5 single high-quality blastocyst	240	145	2/145 (1.4)	0.4–4.9	<0.001	0	2/240 (0.8)	0.2–3.0	<0.001
	D5 double high-quality blastocysts	52	34	14/34 (41.2)	26.4–57.8		0	14/52 (26.9)	16.8–40.3	
	D5 double blastocysts (1 high-quality + 1 usable)	23	15	4/15 (26.7)	10.9–52.0		0	4/23 (17.4)	7.0–37.1	
	D5 single usable blastocyst	22	8	0/8 (0.0)	0.0–32.4		0	0/22 (0.0)	0.0–14.9	
	D3 double high-quality cleavage-stage embryos	170	73	28/73 (38.4)	28.1–49.8		0	28/170 (16.5)	11.6–22.8	
	D3 single high-quality cleavage-stage embryo	18	8	0/8 (0.0)	0.0–32.4		0	0/18 (0.0)	0.0–17.6	
	D6 single high-quality blastocyst	60	31	0/31 (0.0)	0.0–11.0		0	0/60 (0.0)	0.0–6.0	
	D6 double high-quality blastocysts	8	6	1/6 (16.7)	3.0–56.4		0	1/8 (12.5)	2.2–47.1	
	Sequential transfer (two usable embryos)	122	60	17/60 (28.3)	18.5–40.8		0	17/122 (13.9)	8.9–21.2	
35–39 years	D5 single high-quality blastocyst	198	106	5/106 (4.7)	2.0–10.6	<0.001	0	5/198 (2.5)	1.1–5.8	<0.001
	D5 double high-quality blastocysts	66	47	16/47 (34.0)	22.2–48.3		0	16/66 (24.2)	15.5–35.8	
	D5 double blastocysts (1 high-quality + 1 usable)	21	12	5/12 (41.7)	19.3–68.0		0	5/21 (23.8)	10.6–45.1	
	D5 single usable blastocyst	24	8	0/8 (0.0)	0.0–32.4		0	0/24 (0.0)	0.0–13.8	
	D3 double high-quality cleavage-stage embryos	192	88	27/88 (30.7)	22.0–41.0		0	27/192 (14.1)	9.8–19.7	
	D3 single high-quality cleavage-stage embryo	32	9	0/9 (0.0)	0.0–29.9		0	0/32 (0.0)	0.0–10.7	
	D6 single high-quality blastocyst	39	13	0/13 (0.0)	0.0–22.8		0	0/39 (0.0)	0.0–9.0	
	D6 double high-quality blastocysts	8	3	0/3 (0.0)	0.0–56.1		0	0/8 (0.0)	0.0–32.4	
	Sequential transfer (two usable embryos)	139	69	11/69 (15.9)	9.1–26.3		0	11/139 (7.9)	4.5–13.6	
≥40 years	D5 single high-quality blastocyst	39	16	0/16 (0.0)	0.0–19.4	0.063	0	0/39 (0.0)	0.0–9.0	0.007
	D5 double high-quality blastocysts	18	10	4/10 (40.0)	16.8–68.7		0	4/18 (22.2)	9.0–45.2	
	D5 double blastocysts (1 high-quality + 1 usable)	7	3	1/3 (33.3)	6.1–79.2		0	1/7 (14.3)	2.6–51.3	
	D5 single usable blastocyst	7	2	0/2 (0.0)	0.0–65.8		0	0/7 (0.0)	0.0–35.4	
	D3 double high-quality cleavage-stage embryos	133	36	3/36 (8.3)	2.9–21.8		0	3/133 (2.3)	0.8–6.4	
	D3 single high-quality cleavage-stage embryo	30	4	0/4 (0.0)	0.0–49.0		0	0/30 (0.0)	0.0–11.4	
	D6 single high-quality blastocyst	12	4	0/4 (0.0)	0.0–49.0		0	0/12 (0.0)	0.0–24.2	
	D6 double high-quality blastocysts	0	0	—	—		0	—	—	
	Sequential transfer (two usable embryos)	68	15	1/15 (6.7)	1.2–29.8		1	1/68 (1.5)	0.3–7.9	

Triplet pregnancies were reported separately and were not included in the numerator for twin pregnancy. The twin pregnancy rate among clinical pregnancies used the number of clinical pregnancy cycles within each age group and transfer strategy as the denominator; the twin pregnancy risk per transfer cycle used all transfer cycles as the denominator. Ultrasound fetal-number data were available for all clinical pregnancy cycles. The 95% CIs were calculated using the Wilson method. Overall comparisons among the nine strategies within each age group were performed using the Fisher-Freeman-Halton exact test with 200,000 Monte Carlo repetitions. No D6 double high-quality blastocyst transfers occurred in women aged ≥40 years.

### 3.4 Multivariable analyses of clinical pregnancy and live birth

**Figure 2:**
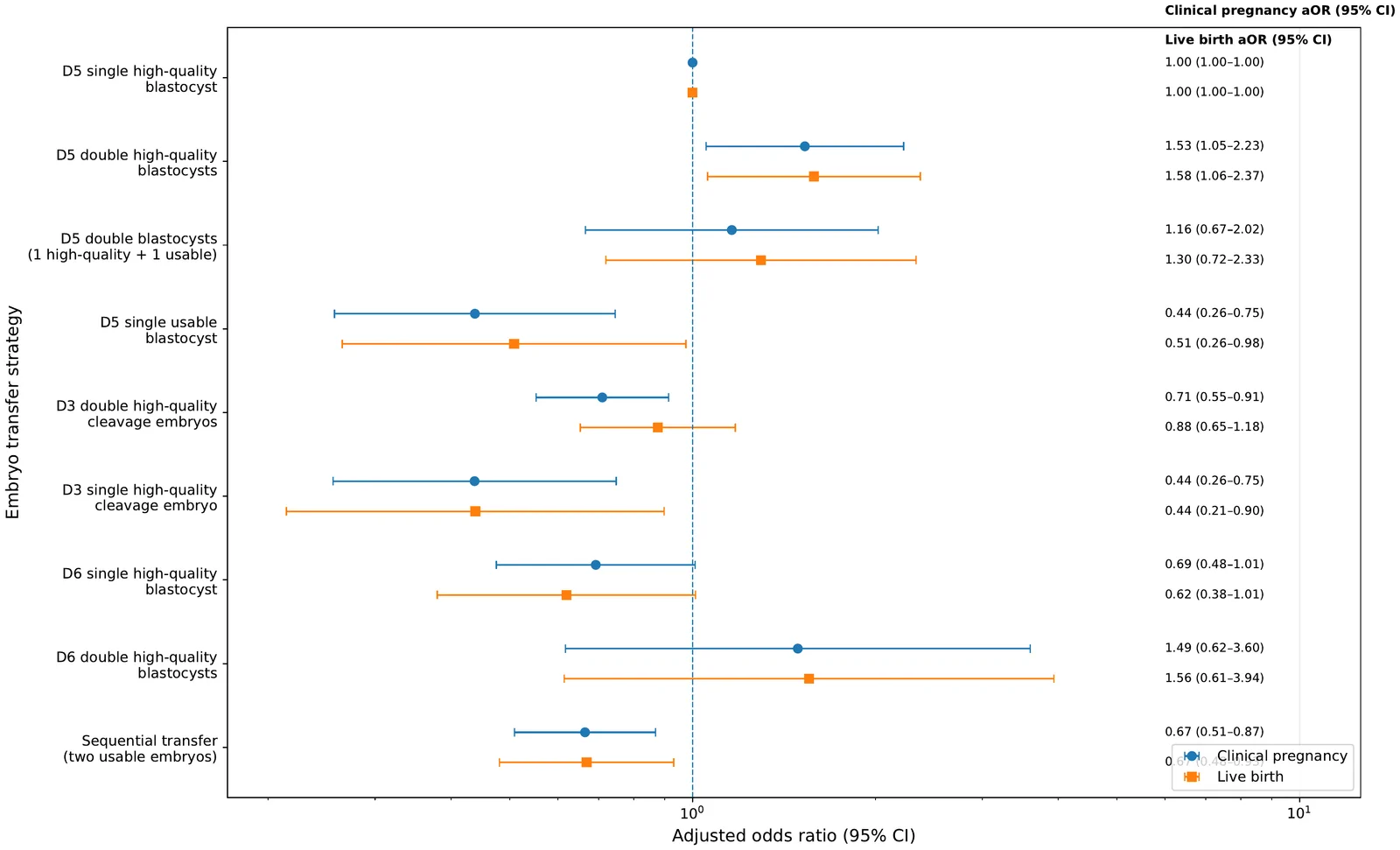
Forest plot of multivariable-adjusted odds ratios for clinical pregnancy and live birth across 9 embryo transfer strategies. D5 single high-quality blastocyst transfer was used as the reference group. Points represent adjusted odds ratios (aORs), and horizontal lines indicate 95% confidence intervals (CIs). The models were adjusted for actual female age, body mass index (BMI), duration of infertility, infertility factor category, polycystic ovary syndrome (PCOS), anti-Müllerian hormone (AMH), number of usable embryos, fertilization method, endometrial preparation regimen, and endometrial thickness on the day of transfer. The clinical pregnancy model included 2,143 transfer cycles, whereas the live birth model included 1,802 cycles with known live birth outcomes.

Using D5 single high-quality blastocyst transfer as the reference, and after adjustment for actual female age, BMI, duration of infertility, infertility factor category, PCOS, AMH, number of usable embryos, fertilization method, endometrial preparation regimen, and endometrial thickness on the day of transfer, D5 double high-quality blastocyst transfer was associated with higher odds of clinical pregnancy (*P*=0.026). D5 single usable blastocyst transfer, D3 double high-quality cleavage-stage embryo transfer, D3 single high-quality cleavage-stage embryo transfer, and sequential transfer were associated with lower odds of clinical pregnancy. No statistically significant differences from the reference strategy were observed for D6 single high-quality blastocyst transfer, D5 double blastocyst transfer comprising one high-quality and one usable blastocyst, or D6 double high-quality blastocyst transfer (**Table 5A**; **Figure 2**).

**Table 5:** Logistic regression analyses of the associations between embryo transfer strategy and clinical pregnancy or live birth.

A. Clinical pregnancy outcome (n = 2,143; 1,053 clinical pregnancies)						
Embryo transfer strategy	Crude model, OR (95% CI)	P value	Model 1, aOR (95% CI)	P value	Model 2, aOR (95% CI)	P value
D5 single high-quality blastocyst	1.00 (reference)	—	1.00 (reference)	—	1.00 (reference)	—
D5 double high-quality blastocysts	1.47 (1.02–2.11)	0.039	1.64 (1.14–2.38)	0.008	1.53 (1.05–2.23)	0.026
D5 double blastocysts (1 high-quality + 1 usable)	1.13 (0.65–1.94)	0.669	1.20 (0.69–2.10)	0.511	1.16 (0.67–2.02)	0.599
D5 single usable blastocyst	0.38 (0.22–0.63)	<0.001	0.39 (0.23–0.65)	<0.001	0.44 (0.26–0.75)	0.002
D3 double high-quality cleavage-stage embryos	0.51 (0.41–0.64)	<0.001	0.61 (0.48–0.77)	<0.001	0.71 (0.55–0.91)	0.008
D3 single high-quality cleavage-stage embryo	0.25 (0.15–0.41)	<0.001	0.33 (0.20–0.56)	<0.001	0.44 (0.26–0.75)	0.003
D6 single high-quality blastocyst	0.59 (0.41–0.85)	0.005	0.62 (0.43–0.90)	0.011	0.69 (0.48–1.01)	0.056
D6 double high-quality blastocysts	1.44 (0.61–3.41)	0.408	1.41 (0.59–3.37)	0.439	1.49 (0.62–3.60)	0.375
Sequential transfer (two usable embryos)	0.57 (0.44–0.74)	<0.001	0.66 (0.51–0.86)	0.002	0.67 (0.51–0.87)	0.003
B. Live birth outcome (n = 1,802; 533 live births; 341 cycles with unknown live birth outcomes excluded)						
Embryo transfer strategy	Crude model, OR (95% CI)	P value	Model 1, aOR (95% CI)	P value	Model 2, aOR (95% CI)	P value
D5 single high-quality blastocyst	1.00 (reference)	—	1.00 (reference)	—	1.00 (reference)	—
D5 double high-quality blastocysts	1.46 (0.99–2.15)	0.057	1.70 (1.14–2.53)	0.009	1.58 (1.06–2.37)	0.025
D5 double blastocysts (1 high-quality + 1 usable)	1.26 (0.71–2.25)	0.426	1.38 (0.77–2.48)	0.282	1.30 (0.72–2.33)	0.388
D5 single usable blastocyst	0.44 (0.23–0.82)	0.010	0.45 (0.24–0.86)	0.016	0.51 (0.26–0.98)	0.042
D3 double high-quality cleavage-stage embryos	0.64 (0.49–0.84)	0.001	0.77 (0.58–1.02)	0.064	0.88 (0.65–1.18)	0.380
D3 single high-quality cleavage-stage embryo	0.25 (0.13–0.50)	<0.001	0.35 (0.17–0.70)	0.003	0.44 (0.21–0.90)	0.024
D6 single high-quality blastocyst	0.54 (0.33–0.87)	0.011	0.55 (0.34–0.90)	0.016	0.62 (0.38–1.01)	0.055
D6 double high-quality blastocysts	1.46 (0.59–3.58)	0.413	1.47 (0.59–3.67)	0.408	1.56 (0.61–3.94)	0.351
Sequential transfer (two usable embryos)	0.56 (0.41–0.76)	<0.001	0.66 (0.48–0.91)	0.012	0.67 (0.48–0.93)	0.017

D5 single high-quality blastocyst transfer was the reference group. The crude model included embryo transfer strategy only. Model 1 adjusted for actual female age, body mass index, duration of infertility, infertility factor category, and PCOS. Model 2 additionally adjusted for AMH, number of usable embryos, fertilization method, endometrial preparation regimen, and endometrial thickness on the day of transfer. The clinical pregnancy analysis included all 2,143 cycles. The live birth analysis included 1,802 cycles with known outcomes and excluded 341 cycles with unknown live birth outcomes. OR, odds ratio; aOR, adjusted odds ratio; CI, confidence interval; AMH, anti-Müllerian hormone; PCOS, polycystic ovary syndrome.

Among the 1,802 cycles with known live birth outcomes, D5 double high-quality blastocyst transfer was associated with higher odds of live birth. D5 single usable blastocyst transfer, D3 single high-quality cleavage-stage embryo transfer, and sequential transfer were associated with lower odds of live birth. The associations of D3 double high-quality cleavage-stage embryo transfer and D6 single high-quality blastocyst transfer with live birth were no longer statistically significant after full adjustment (**Table 5B**; **Figure 2**).

### 3.5 Firth logistic regression analysis of per-transfer-cycle twin pregnancy risk

Because no twin pregnancy events occurred in the D5 single usable blastocyst group or the D3 single high-quality cleavage-stage embryo group, Firth bias-reduced logistic regression was applied. After exclusion of the three triplet pregnancies, the primary analysis included 2,140 transfer cycles, of which 174 resulted in twin pregnancy. Using D5 single high-quality blastocyst transfer as the reference and adjusting for actual female age, BMI, infertility factor category, PCOS, AMH, number of usable embryos, and endometrial preparation regimen, D5 double high-quality blastocyst transfer, D5 double blastocyst transfer comprising one high-quality and one usable blastocyst, D3 double high-quality cleavage-stage embryo transfer, D6 double high-quality blastocyst transfer, and sequential transfer were all associated with a significantly increased per-transfer-cycle risk of twin pregnancy (all *P*<0.001). No statistically significant differences from the reference strategy were observed for D5 single usable blastocyst transfer, D3 single high-quality cleavage-stage embryo transfer, or D6 single high-quality blastocyst transfer. The sensitivity analysis restricted to clinical pregnancy cycles yielded results in the same direction, with a significant overall effect of transfer strategy (*P*<0.001) (**Table 6**).

**Table 6:** Firth logistic regression analyses of the association between embryo transfer strategy and twin pregnancy risk.

Embryo transfer strategy	Twin pregnancy, n/N (%)	Crude model, OR (95% CI)	P value	Adjusted model, aOR (95% CI)	P value	Sensitivity analysis restricted to clinical pregnancy cycles, aOR (95% CI)	P value
D5 single high-quality blastocyst	8/650 (1.2)	1.00 (reference)	—	1.00 (reference)	—	1.00 (reference)	—
D5 double high-quality blastocysts	42/161 (26.1)	26.88 (12.54–57.65)	<0.001	32.67 (15.21–70.19)	<0.001	33.76 (15.32–74.42)	<0.001
D5 double blastocysts (1 high-quality + 1 usable)	11/59 (18.6)	17.92 (7.02–45.77)	<0.001	19.38 (7.57–49.61)	<0.001	21.63 (8.04–58.17)	<0.001
D5 single usable blastocyst	0/70 (0.0)	0.54 (0.03–9.57)	0.640	0.57 (0.03–9.94)	0.680	0.96 (0.05–17.79)	0.979
D3 double high-quality cleavage-stage embryos	73/573 (12.7)	11.12 (5.41–22.86)	<0.001	13.45 (6.45–28.05)	<0.001	20.56 (9.70–43.57)	<0.001
D3 single high-quality cleavage-stage embryo	0/86 (0.0)	0.44 (0.02–7.76)	0.521	0.61 (0.03–10.74)	0.720	1.08 (0.06–20.42)	0.960
D6 single high-quality blastocyst	2/142 (1.4)	1.34 (0.32–5.60)	0.691	1.47 (0.36–6.04)	0.611	1.96 (0.47–8.28)	0.389
D6 double high-quality blastocysts	4/24 (16.7)	16.59 (4.79–57.46)	<0.001	16.39 (4.72–56.91)	<0.001	14.24 (3.85–52.62)	<0.001
Sequential transfer (two usable embryos)	34/378 (9.0)	7.61 (3.55–16.32)	<0.001	9.49 (4.43–20.36)	<0.001	13.12 (6.01–28.67)	<0.001
Overall effect of transfer strategy			<0.001		<0.001		<0.001

The primary analysis included all transfer cycles, whereas the sensitivity analysis was restricted to clinical pregnancy cycles. The n/N (%) values were calculated using all 2,143 transfer cycles as the denominator. After excluding three triplet pregnancies, the primary regression analysis included 2,140 cycles, including 174 twin pregnancies. The sensitivity analysis included 1,050 clinical pregnancy cycles, including 174 twin pregnancies. The adjusted model included embryo transfer strategy, actual female age, body mass index, infertility factor category, PCOS, AMH, number of usable embryos, and endometrial preparation regimen. Because no twin pregnancy events occurred in the D5 single usable blastocyst and D3 single high-quality cleavage-stage embryo groups, Firth bias-reduced logistic regression was used. The 95% CIs were based on the penalized information matrix, and P values were obtained using penalized likelihood-ratio tests. OR, odds ratio; aOR, adjusted odds ratio; CI, confidence interval; AMH, anti-Müllerian hormone; PCOS, polycystic ovary syndrome.

### 3.6 Age-by-strategy interaction and sensitivity analyses

Age-by-transfer-strategy interaction analyses showed no statistically significant interaction for either clinical pregnancy or live birth (**Table 7A**). In sensitivity analyses, replacing AMH with AFC or replacing the overall infertility factor category with four specific infertility factors produced associations that were generally consistent in direction and magnitude with those observed in the primary models. After exclusion of women with PCOS, the clinical pregnancy findings remained consistent, and the overall effect of transfer strategy remained statistically significant (*P*<0.001). The overall effect of transfer strategy on live birth also remained significant (*P*=0.007), although the confidence intervals for some individual comparisons crossed 1 because of the reduced sample size. When all 341 cycles with unknown live birth outcomes were conservatively classified as no live birth, D5 double high-quality blastocyst transfer remained associated with higher odds of live birth (aOR=1.60, 95% CI: 1.10–2.33). The associations for several other strategies were attenuated, but the overall effect of transfer strategy remained statistically significant (*P*<0.001) (**Table 7B**).

**Table 7:** Interaction and sensitivity analyses of female age and embryo transfer strategy.

A. Interaction tests between female age and embryo transfer strategy										
Outcome		Interaction term	Sample size		Events, n	df		Likelihood-ratio χ²		P for interaction
Clinical pregnancy		Actual age (continuous) × transfer strategy	2143		1053	8		6.05		0.641
Clinical pregnancy		Age group (4 categories) × transfer strategy	2143		1053	23		17.99		0.758
Live birth		Actual age (continuous) × transfer strategy	1802		533	8		5.6		0.691
Live birth		Age group (4 categories) × transfer strategy	1802		533	23		18.48		0.731
B. Effects of embryo transfer strategy in prespecified sensitivity analyses										
Outcome/analysis	Sample size/events	D5 double high-quality blastocysts	D5 double blastocysts (1 high-quality + 1 usable)	D5 single usable blastocyst	D3 double high-quality cleavage-stage embryos	D3 single high-quality cleavage-stage embryo	D6 single high-quality blastocyst	D6 double high-quality blastocysts	Sequential transfer (two usable embryos)	Overall P value
Clinical pregnancy: primary model	2143/1053	1.53 (1.05–2.23)	1.16 (0.67–2.02)	0.44 (0.26–0.75)	0.71 (0.55–0.91)	0.44 (0.26–0.75)	0.69 (0.48–1.01)	1.49 (0.62–3.60)	0.67 (0.51–0.87)	<0.001
Clinical pregnancy: AFC substituted for AMH	2143/1053	1.53 (1.05–2.23)	1.16 (0.67–2.03)	0.44 (0.26–0.75)	0.71 (0.55–0.91)	0.44 (0.26–0.75)	0.69 (0.48–1.01)	1.49 (0.62–3.61)	0.67 (0.51–0.87)	<0.001
Clinical pregnancy: four specific infertility factors substituted for infertility factor category	2143/1053	1.57 (1.08–2.28)	1.21 (0.69–2.11)	0.43 (0.25–0.73)	0.71 (0.55–0.92)	0.44 (0.25–0.75)	0.70 (0.48–1.03)	1.50 (0.62–3.64)	0.66 (0.51–0.87)	<0.001
Clinical pregnancy: patients with PCOS excluded	1762/828	1.64 (1.07–2.49)	1.31 (0.69–2.49)	0.49 (0.28–0.87)	0.69 (0.52–0.91)	0.43 (0.25–0.76)	0.85 (0.56–1.27)	1.56 (0.56–4.39)	0.66 (0.49–0.88)	<0.001
Live birth: primary model	1802/533	1.58 (1.06–2.37)	1.30 (0.72–2.33)	0.51 (0.26–0.98)	0.88 (0.65–1.18)	0.44 (0.21–0.90)	0.62 (0.38–1.01)	1.56 (0.61–3.94)	0.67 (0.48–0.93)	<0.001
Live birth: AFC substituted for AMH	1802/533	1.60 (1.07–2.40)	1.33 (0.74–2.40)	0.50 (0.26–0.96)	0.87 (0.65–1.16)	0.43 (0.21–0.88)	0.63 (0.38–1.02)	1.56 (0.61–3.98)	0.67 (0.48–0.93)	<0.001
Live birth: four specific infertility factors substituted for infertility factor category	1802/533	1.54 (1.03–2.30)	1.34 (0.75–2.41)	0.51 (0.26–0.97)	0.88 (0.66–1.18)	0.45 (0.22–0.92)	0.62 (0.38–1.02)	1.51 (0.60–3.81)	0.66 (0.48–0.92)	<0.001
Live birth: patients with PCOS excluded	1500/419	1.57 (0.99–2.48)	1.51 (0.76–2.98)	0.52 (0.26–1.06)	0.84 (0.61–1.17)	0.43 (0.20–0.92)	0.78 (0.47–1.29)	1.62 (0.54–4.91)	0.68 (0.47–0.99)	0.007
Live birth: unknown live birth outcomes coded as no live birth	2143/533	1.60 (1.10–2.33)	1.62 (0.92–2.86)	0.67 (0.35–1.27)	1.05 (0.79–1.40)	0.54 (0.26–1.09)	0.64 (0.40–1.02)	1.65 (0.70–3.92)	0.74 (0.54–1.02)	<0.001

All models used D5 single high-quality blastocyst transfer as the reference group. The primary model adjusted for actual female age, body mass index, duration of infertility, infertility factor category, PCOS, AMH, number of usable embryos, fertilization method, endometrial preparation regimen, and endometrial thickness on the day of transfer. Interactions were assessed using likelihood-ratio tests comparing nested logistic regression models, with age entered either as a continuous variable or as four age groups. Sensitivity analyses included replacing AMH with AFC, replacing the overall infertility factor category with four specific infertility factors, excluding patients with PCOS, and coding all 341 cycles with unknown live birth outcomes as no live birth. The primary live birth analysis included 1,802 cycles with known outcomes, whereas the conservative sensitivity analysis included all 2,143 cycles. No D6 double high-quality blastocyst transfers occurred in women aged ≥40 years. Values in part B are aORs (95% CIs). aOR, adjusted odds ratio; CI, confidence interval; AMH, anti-Müllerian hormone; AFC, antral follicle count; PCOS, polycystic ovary syndrome.

### 3.7 Age-stratified adjusted predicted probabilities

**Figure 3:**
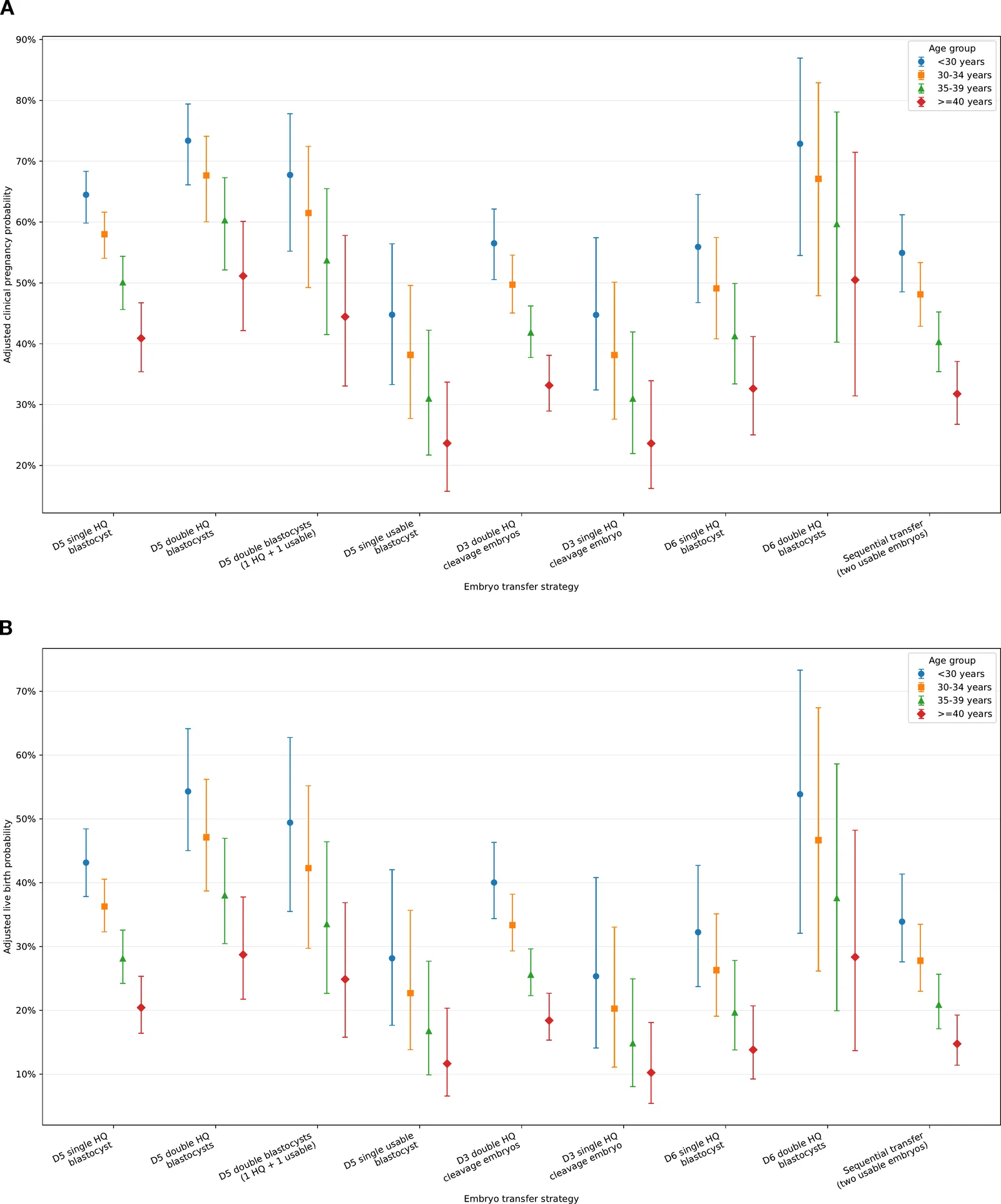
**(A)** Age-stratified adjusted predicted probabilities of clinical pregnancy. Age was categorized as <30, 30–34, 35–39, and ≥40 years. The vertical axis represents the multivariable-adjusted predicted probability of clinical pregnancy derived from the multivariable logistic regression model. Points indicate predicted probabilities, and error bars represent 95% CIs. The model was adjusted for the same covariates as Model 2 in **Table 5**. HQ, high-quality.In the “1 HQ + 1 usable” group, “usable” refers to a non-high-quality usable blastocyst. **(B)** Age-stratified adjusted predicted probabilities of live birth. Age was categorized as <30, 30–34, 35–39, and ≥40 years. The vertical axis represents the multivariable-adjusted predicted probability of live birth derived from the multivariable logistic regression model. Points indicate predicted probabilities, and error bars represent 95% CIs. The live birth analysis included 1,802 transfer cycles with known live birth outcomes, and the model was adjusted for the same covariates as Model 2 in **Table 5**.HQ, high-quality.In the “1 HQ + 1 usable” group, “usable” refers to a non-high-quality usable blastocyst.

In the fully adjusted main-effects models, the predicted probabilities of clinical pregnancy and live birth generally decreased with increasing age across all transfer strategies. Further age-by-transfer-strategy interaction tests were not statistically significant. Because no D6 double high-quality blastocyst transfer cycles occurred among women aged ≥40 years, the predicted value for this age–strategy combination was based on model extrapolatio and should therefore be interpreted with caution (**Figures 3A, B**; **Supplementary Figures 1, 2**).

## 4 Discussion

Using real-world cohort data, this study systematically compared nine embryo transfer strategies defined by embryo developmental stage, embryo quality, and the number of embryos transferred, and further evaluated the potential modifying effect of female age on the associations of these strategies with clinical pregnancy and live birth. The principal findings showed that D5 single high-quality blastocyst transfer achieved favorable clinical pregnancy rates across age groups while maintaining a relatively low per-transfer-cycle risk of twin pregnancy. D5 double high-quality blastocyst transfer was associated with higher odds of clinical pregnancy and live birth, but also with a markedly increased per-transfer-cycle risk of twin pregnancy. The outcomes of several D3 cleavage-stage embryo, D6 blastocyst, and sequential transfer strategies were inferior to, or not significantly better than, those of D5 single high-quality blastocyst transfer. No statistically significant age-by-transfer-strategy interaction was observed, suggesting that the available data were insufficient to demonstrate that the relative effects of the transfer strategies varied substantially with female age. Therefore, selection of an embryo transfer strategy should incorporate female age, embryo developmental stage and quality, the number of usable embryos, and the risk of multiple pregnancy, rather than being based solely on the unadjusted pregnancy rate within a particular age group.

### 4.1 D5 single high-quality blastocyst transfer: a reference strategy balancing reproductive outcomes and multiple-pregnancy risk

D5 single high-quality blastocyst transfer was the most frequently used strategy in this study, and its clinical pregnancy rate generally declined with increasing female age. The twin pregnancy rates among clinical pregnancies across the four age groups were 0.9%, 1.4%, 4.7%, and 0, respectively, while the corresponding per-transfer-cycle twin pregnancy risks were 0.6%, 0.8%, 2.5%, and 0. These findings indicate that this strategy was associated with a generally low risk of multiple pregnancy, although twin pregnancy was not completely eliminated. This observation is consistent with current international guidelines advocating elective single-blastocyst transfer (eSET) to reduce multiple pregnancy and its associated maternal and neonatal complications (10). Although some studies have suggested that embryo developmental stage may have a limited effect on reproductive outcomes, most available evidence indicates that blastocyst transfer may provide greater implantation potential and a higher probability of live birth when a usable high-quality blastocyst is available (12, 13). Previous studies have also shown that, among young women with a favorable prognosis, single high-quality blastocyst transfer can maintain satisfactory reproductive outcomes while reducing the risks of multiple pregnancy and preterm birth (4, 10).

In the present study, women aged <35 years who underwent D5 single high-quality blastocyst transfer achieved a clinical pregnancy rate exceeding 60%, while the per-transfer-cycle twin pregnancy risk remained below 1%, suggesting that this strategy may provide a favorable balance between effectiveness and safety. Although the clinical pregnancy and live birth rates decreased to 53.5% and 27.4%, respectively, among women aged 35–39 years, the per-transfer-cycle twin pregnancy risk remained relatively low at 2.5%. Previous research has similarly shown that single high-quality blastocyst transfer can maintain a low twin pregnancy rate in this age group, substantially below that observed after double-embryo transfer (8). Among women aged ≥40 years, although the unadjusted clinical pregnancy and live birth rates were relatively high in the D5 double high-quality blastocyst group, the per-transfer-cycle twin pregnancy risk was also markedly increased, and the number of cycles was limited. Therefore, decisions regarding whether to increase the number of embryos transferred in women of advanced reproductive age should take into account embryo quality, previous treatment history, patient preferences, and the risk of multiple pregnancy.

### 4.2 D5 double high-quality blastocyst transfer: potential pregnancy benefit accompanied by increased twin pregnancy risk

Compared with D5 single high-quality blastocyst transfer, D5 double high-quality blastocyst transfer remained associated with higher odds of clinical pregnancy and live birth after full adjustment, suggesting that the addition of a second high-quality D5 blastocyst may increase the probability of achieving pregnancy and live birth within a single transfer cycle. However, this potential benefit was accompanied by a marked increase in the per-transfer-cycle risk of twin pregnancy. Firth logistic regression showed that the adjusted odds of twin pregnancy in the D5 double high-quality blastocyst group were approximately 33-fold higher than those in the reference group. The direction of the association remained consistent in the sensitivity analysis restricted to clinical pregnancy cycles.

Previous studies have likewise shown that double-embryo transfer may increase the probability of pregnancy within a single cycle but substantially increases the risks of multiple pregnancy and associated maternal and neonatal complications (3, 4, 14). The present findings further emphasize that any potential reproductive benefit obtained by increasing the number of embryos transferred should not be evaluated independently of the accompanying risk of twin pregnancy. Notably, no significant interaction between female age and transfer strategy was observed for either clinical pregnancy or live birth. Therefore, the unadjusted outcome rates in a particular age group do not provide sufficient evidence to conclude that D5 double high-quality blastocyst transfer is appropriate only for women of advanced reproductive age or that its per-transfer-cycle twin pregnancy risk consistently decreases with increasing age. For patients considering double high-quality blastocyst transfer, individualized decision-making should be based on comprehensive counseling regarding the potential benefit within a single transfer cycle, the risk of twin pregnancy, and the subsequent obstetric risks.

### 4.3 Addition of one non-high-quality usable blastocyst did not confer a clear reproductive benefit

Transfer of two D5 blastocysts comprising one high-quality blastocyst and one non-high-quality usable blastocyst is a relatively common clinical strategy. Compared with D5 single high-quality blastocyst transfer, this strategy showed no statistically significant advantage in either the adjusted clinical pregnancy or live birth model but was associated with a markedly increased per-transfer-cycle risk of twin pregnancy. Previous studies comparing the transfer of a single high-quality embryo with the transfer of a high-quality embryo plus one low-quality embryo have shown that the additional contribution of the second low-quality embryo to live birth is inconsistent, whereas the risks of multiple pregnancy and adverse neonatal outcomes may be substantially increased (15). Similarly, the present findings suggest that, when one high-quality D5 blastocyst is already available, the addition of a non-high-quality usable blastocyst should be considered cautiously and should not be routinely undertaken solely to increase the probability of success within a single transfer cycle.

### 4.4 D3 high-quality cleavage-stage embryos: continued clinical value without an overall advantage for double-embryo transfer

Compared with D5 single high-quality blastocyst transfer, D3 double high-quality cleavage-stage embryo transfer was associated with lower odds of clinical pregnancy after full adjustment, whereas the difference in live birth was not statistically significant. These findings suggest that D3 double high-quality cleavage-stage embryo transfer may be less favorable than D5 single high-quality blastocyst transfer in terms of early pregnancy outcomes. However, the unadjusted difference in live birth was attenuated after controlling for age, ovarian reserve, embryo availability, and endometrial factors, indicating that part of the observed difference may be attributable to patient selection and baseline prognosis. Previous studies have suggested that extended culture of thawed D3 embryos to the blastocyst stage may improve pregnancy outcomes in some patients with recurrent implantation failure, while embryo developmental stage and the endometrial preparation regimen may both influence transfer outcomes in women of advanced reproductive age (11, 16).

It should also be noted that the per-transfer-cycle risk of twin pregnancy was markedly higher after D3 double high-quality cleavage-stage embryo transfer than after D5 single high-quality blastocyst transfer. Therefore, when both D5 high-quality blastocysts and D3 high-quality cleavage-stage embryos are available, the present findings do not indicate a more favorable effectiveness–safety balance for D3 double-embryo transfer. Nevertheless, D3 high-quality cleavage-stage embryos remain a clinically usable resource for patients who do not obtain blastocysts or are not suitable candidates for extended blastocyst culture, and the present results should not be interpreted as indicating that D3 embryos have no clinical value.

D3 single high-quality cleavage-stage embryo transfer was associated with lower odds of both clinical pregnancy and live birth. However, this group had a relatively small sample size, and no twin pregnancy events were observed. The less favorable outcomes may have been related to embryo developmental stage, but they may also reflect poorer embryo availability and baseline prognosis among patients selected for single D3 embryo transfer. Accordingly, these results should primarily be interpreted as observational associations rather than as evidence of a direct adverse effect of transferring a single D3 embryo.

### 4.5 D6 blastocyst transfer: uncertainty regarding effectiveness and twin pregnancy risk

Compared with D5 single high-quality blastocyst transfer, D6 single high-quality blastocyst transfer yielded adjusted odds ratios below 1 for both clinical pregnancy and live birth; however, the 95% CIs crossed 1, and neither association reached statistical significance. No marked increase in the per-transfer-cycle risk of twin pregnancy was observed. These findings suggest that D6 single high-quality blastocyst transfer may represent an option associated with a relatively low risk of multiple pregnancy, although the available data are insufficient to determine whether its reproductive outcomes are comparable to those of D5 single high-quality blastocyst transfer. A previous age-stratified study comparing D6 high-quality blastocysts with D5 non-high-quality blastocysts likewise found no clear clinical advantage for D6 high-quality blastocysts, indicating that the day of blastocyst development and morphological quality should be evaluated jointly (17).D6 double high-quality blastocyst transfer showed no clear advantage in clinical pregnancy or live birth but was associated with a markedly increased per-transfer-cycle risk of twin pregnancy. Because this group included only 24 cycles and no such transfers occurred among women aged ≥40 years, the estimates were imprecise and accompanied by wide confidence intervals. Previous evidence has suggested that live birth outcomes after D6 blastocyst transfer in frozen-thawed cycles may be inferior to those after D5 blastocyst transfer, although the results may also be influenced by cycle type, embryo quality, and patient characteristics (18). Therefore, considerable uncertainty remains regarding the effectiveness of D6 double high-quality blastocyst transfer. The absence of a clear reproductive benefit, together with the observed increase in twin pregnancy risk, indicates that increasing the number of D6 blastocysts transferred should be approached cautiously.

### 4.6 Sequential transfer was not superior to D5 single high-quality blastocyst transfer

Sequential transfer involves transferring a D3 cleavage-stage embryo followed by a D5 or D6 blastocyst within the same treatment cycle and may theoretically increase the likelihood of synchrony between embryo development and the implantation window. In the present study, however, sequential transfer was associated with lower odds of clinical pregnancy and live birth after adjustment, while the per-transfer-cycle risk of twin pregnancy was markedly higher than that observed with D5 single high-quality blastocyst transfer.

These findings do not support the routine use of sequential transfer to improve FET outcomes. Previous studies of sequential transfer have mainly focused on patients with recurrent implantation failure. Although some studies have reported improvements in clinical pregnancy outcomes, the available findings remain inconsistent, and the populations investigated differ from that of the present study (19). The less favorable outcomes observed in our cohort may reflect the more complex infertility profiles, previous treatment histories, or embryo-resource characteristics of patients selected for sequential transfer. Moreover, transferring additional embryos may increase the probability of multiple implantations without producing a corresponding improvement in the likelihood of live birth. Although the multivariable models adjusted for several potential confounders, confounding by indication cannot be completely excluded. The clinical value of sequential transfer should therefore be further evaluated in prospective studies involving patients with clearly defined indications, rather than being considered a routine alternative strategy on the basis of the present findings.

### 4.7 Limitations

This study has several limitations. First, the single-center retrospective design could not fully eliminate selection bias or confounding by indication. The transfer strategies were not randomly assigned, and residual confounding related to previous transfer history, clinician preference, and unmeasured embryo characteristics may have persisted despite multivariable adjustment. Second, some transfer strategies and age–strategy combinations included relatively few cycles. In particular, only 24 cycles involved D6 double high-quality blastocyst transfer, and no such transfers occurred among women aged ≥40 years, limiting the precision of the age-interaction analyses and age-specific estimates. Third, live birth outcomes were unknown for 341 cycles. Although a conservative sensitivity analysis was performed, loss-to-follow-up bias remains possible. Fourth, cumulative live birth, time to live birth, preterm birth, low birth weight, and other maternal and neonatal outcomes were not evaluated. Consequently, the long-term overall benefits and risks of the different strategies could not be fully assessed. Previous studies have suggested that embryo developmental stage and multiple pregnancy may be associated with perinatal outcomes (20, 21). Further multicenter prospective studies are therefore warranted.

## 5 Conclusions

Clinical pregnancy, live birth, and per-transfer-cycle twin pregnancy risk differed across frozen-thawed embryo transfer strategies. D5 single high-quality blastocyst transfer generally yielded favorable reproductive outcomes and a relatively low per-transfer-cycle risk of twin pregnancy and may therefore serve as a clinically relevant reference strategy. D5 double high-quality blastocyst transfer may increase the probability of clinical pregnancy and live birth within a single transfer cycle but also substantially increases the per-transfer-cycle risk of twin pregnancy. Adding one non-high-quality usable blastocyst when a D5 high-quality blastocyst is already available did not confer a clear benefit in clinical pregnancy or live birth and was accompanied by an increased twin pregnancy risk. Compared with D5 single high-quality blastocyst transfer, D3 double high-quality cleavage-stage embryo transfer was associated with lower odds of clinical pregnancy and a higher risk of twin pregnancy. D6 double high-quality blastocyst transfer showed no clear advantage in clinical pregnancy or live birth but was associated with an increased twin pregnancy risk. Sequential transfer was associated with lower odds of clinical pregnancy and live birth and a higher risk of twin pregnancy. No statistically significant age-by-transfer-strategy interaction was identified; therefore, the present findings do not support defining fixed transfer-strategy thresholds based solely on female age. Clinical decisions should be individualized by considering baseline patient prognosis, embryo developmental stage and quality, the number of usable embryos, and the risk of multiple pregnancy.

## Data Availability

The raw data supporting the conclusions of this article will be made available by the authors, without undue reservation.

## References

[1] Zhou Y Ji H Zhang M Zhang J Li X Zhang J . Single versus double blastocyst transfer in first and second frozen-thawed embryo transfer cycle in advance-aged women: a two-center retrospective cohort study. BMC Womens Health. (2024) 24:51. doi: 10.1186/s12905-023-02753-x 38238733 PMC10795208

[2] Jiang J Wu X Sun H Han L Zhang X Liu C . Effects of various transfer strategies of frozen-thawed cleavage-stage and blastocyst embryos on pregnancy and neonatal outcomes in different age groups. J Multidiscip Healthc. (2025) 18:2319–34. doi: 10.2147/JMDH.S502766 40302817 PMC12039847

[3] Wang Z Zhu H Tong X Jiang L Wei Q Zhang S . Clinical outcomes after elective double-embryo transfer in frozen cycles for women of advanced maternal age: a retrospective cohort study. Med (Baltimore). (2022) 101:e28992. doi: 10.1097/MD.0000000000028992 35244074 PMC8896420

[4] Wu X Zhou WJ Xu BF Chen Q Xia L Zhao S . Association between transferred embryos and multiple pregnancy/live birth rate in frozen embryo transfer cycles: a retrospective study. Front Endocrinol (Lausanne). (2023) 13:1073164. doi: 10.3389/fendo.2022.1073164 36686447 PMC9849691

[5] Alpha Scientists in Reproductive Medicine ESHRE Special Interest Group of Embryology . The Istanbul consensus workshop on embryo assessment: proceedings of an expert meeting. Hum Reprod. (2011) 26:1270–83. doi: 10.1093/humrep/der037 21502182

[6] Gardner DK Schoolcraft WB . Culture and transfer of human blastocysts. Curr Opin Obstet Gynecol. (1999) 11:307–11. doi: 10.1097/00001703-199906000-00013 10369209

[7] Gardner DK Lane M Stevens J Schlenker T Schoolcraft WB . Blastocyst score affects implantation and pregnancy outcome: towards a single blastocyst transfer. Fertil Steril. (2000) 73:1155–8. doi: 10.1016/S0015-0282(00)00518-5 10856474

[8] Wu Y Lu X Chen H Fu Y Zhao J . Comparison of frozen-thaw blastocyst transfer strategies in women aged 35-40 years: a retrospective study. Front Endocrinol (Lausanne). (2023) 14:1141605. doi: 10.3389/fendo.2023.1141605 37404307 PMC10315647

[9] Zhang X Ma C Wu Z Tao L Li R Liu P . Frozen-thawed embryo transfer cycles have a lower incidence of ectopic pregnancy compared with fresh embryo transfer cycles. Reprod Sci. (2018) 25:1431–5. doi: 10.1177/1933719117746759 29254433

[10] Practice Committee of the American Society for Reproductive Medicine Practice Committee for the Society for Assisted Reproductive Technology . Guidance on the limits to the number of embryos to transfer: a committee opinion. Fertil Steril. (2021) 116:651–4. doi: 10.1016/j.fertnstert.2021.06.050 34330423

[11] Li X Zeng Y He J Luo B Lu X Zhu L . The optimal frozen embryo transfer strategy for the recurrent implantation failure patient without blastocyst freezing: thawing day 3 embryos and culturing to day 5 blastocysts. Zygote. (2023) 31:596–604. doi: 10.1017/S0967199423000503 37969109

[12] Cornelisse S Fleischer K van der Westerlaken L de Bruin JP Vergouw C Koks C . Cumulative live birth rate of a blastocyst versus cleavage stage embryo transfer policy during *in vitro* fertilisation in women with a good prognosis: multicentre randomised controlled trial. BMJ. (2024) 386:e080133. doi: 10.1136/bmj-2024-080133 39284610 PMC11403767

[13] Glujovsky D Cornelisse S Blake D Quinteiro Retamar AM Alvarez Sedo CR Ciapponi A . Blastocyst-stage versus cleavage-stage embryo transfer in assisted reproductive technology. Cochrane Database Syst Rev. (2026) 1:CD002118. doi: 10.1002/14651858.CD002118.pub7 41574757 PMC12828884

[14] Guo J Zeng Z Li M Huang J Peng J Wang M . Clinical outcomes after single-versus double-embryo transfers in women with adenomyosis: a retrospective study. Arch Gynecol Obstet. (2021) 304:263–70. doi: 10.1007/s00404-020-05924-5 33386415

[15] Zeng C Lu RH Li X Wang S Kuai YR Xue Q . Effect of frozen-thawed embryo transfer with a poor-quality embryo and a good-quality embryo on pregnancy and neonatal outcomes. Reprod Biol Endocrinol. (2024) 22:26. doi: 10.1186/s12958-024-01194-x 38383391 PMC10880350

[16] Liu J Zheng J Lei YL Wen XF . Effects of endometrial preparations and transferred embryo types on pregnancy outcome from patients with advanced maternal age. Syst Biol Reprod Med. (2019) 65:181–6. doi: 10.1080/19396368.2018.1501114 30091374

[17] He Y Tang Y Liu H Liu J Mao Y . No advantage of single day 6 good-quality blastocyst transfer versus single day 5 poor-quality blastocyst transfer in frozen-thawed cycles stratified by age: a retrospective study. BMC Pregnancy Childbirth. (2023) 23:79. doi: 10.1186/s12884-023-05387-x 36717810 PMC9885555

[18] Yin B Li S Sun L Yao Z Cui Y Zhang C . Comparing Day 5 versus Day 6 euploid blastocyst in frozen embryo transfer and developing a predictive model for optimizing outcomes: a retrospective cohort study. Front Endocrinol (Lausanne). (2024) 14:1302194. doi: 10.3389/fendo.2023.1302194 38239982 PMC10794779

[19] Gao J Yuan Y Li J Tian T Lian Y Liu P . Sequential embryo transfer versus double cleavage-stage embryo or double blastocyst transfer in patients with recurrent implantation failure with frozen-thawed embryo transfer cycles: a cohort study. Front Endocrinol (Lausanne). (2023) 14:1238251. doi: 10.3389/fendo.2023.1238251 37745696 PMC10515716

[20] Siristatidis C Papapanou M Karageorgiou V Martins WP Bellos I Teixeira DM . Congenital anomaly and perinatal outcome following blastocyst- vs cleavage-stage embryo transfer: systematic review and network meta-analysis. Ultrasound Obstet Gynecol. (2023) 61:12–25. doi: 10.1002/uog.26019 35751886 PMC10107888

[21] Marleen S Kodithuwakku W Nandasena R Mohideen S Allotey J Fernández-García S . Maternal and perinatal outcomes in twin pregnancies following assisted reproduction: a systematic review and meta-analysis involving 802,462 pregnancies. Hum Reprod Update. (2024) 30:309–22. doi: 10.1093/humupd/dmae002 38345641 PMC11063550

