# Supplementary material for: Clinical pregnancy, live birth, and twin pregnancy outcomes of nine frozen-thawed embryo transfer strategies across female age groups: a real-world cohort study
Source: Front Endocrinol (Lausanne). 2026 Sep 1;17:1890802. doi: 10.3389/fendo.2026.1890802 (PMC13574578; doi:10.3389/fendo.2026.1890802)

Supplementary heatmap. Adjusted clinical pregnancy probability

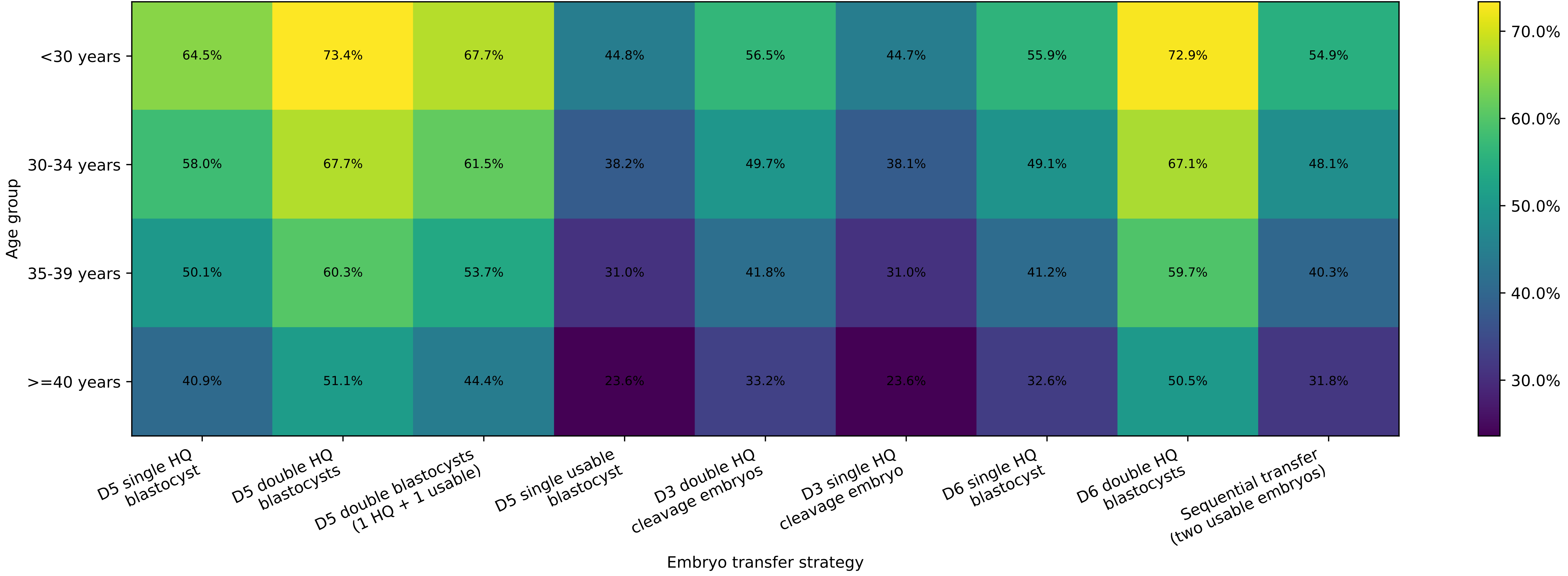

Supplement: Supplementary file 1 [file DataSheet1.pdf]
